# Reconfigurable Dual Peptide Tethered Polymer System Offers a Synergistic Solution for Next Generation Dental Adhesives

**DOI:** 10.3390/ijms22126552

**Published:** 2021-06-18

**Authors:** Esra Yuca, Sheng-Xue Xie, Linyong Song, Kyle Boone, Nilan Kamathewatta, Sarah K. Woolfolk, Philip Elrod, Paulette Spencer, Candan Tamerler

**Affiliations:** 1 IBER Institute for Bioengineering Research, University of Kansas (KU), 1530 W. 15th St, Lawrence, KS 66045, USA; eyuca@ku.edu (E.Y.); sxie@ku.edu (S.-X.X.); leonsong@ku.edu (L.S.); k097b443@ku.edu (K.B.); nilan_jayabahu@ku.edu (N.K.); sarah.vanoosten@ku.edu (S.K.W.); pspencer@ku.edu (P.S.); 2 Department of Molecular Biology and Genetics, Yildiz Technical University, 34210 Istanbul, Turkey; 3 Bioengineering Program, University of Kansas (KU), 1530 W. 15th St, Lawrence, KS 66045, USA; philip.m.elrod@ku.edu; 4 Department of Mechanical Engineering, University of Kansas (KU), 1530 W. 15th St, Lawrence, KS 66045, USA

**Keywords:** biohybrid, biomimetic, bioactive, peptide tethering, antimicrobial peptides, mineralization peptides, dental adhesives, reconfigurable, remineralization

## Abstract

Resin-based composite materials have been widely used in restorative dental materials due to their aesthetic, mechanical, and physical properties. However, they still encounter clinical shortcomings mainly due to recurrent decay that develops at the composite-tooth interface. The low-viscosity adhesive that bonds the composite to the tooth is intended to seal this interface, but the adhesive seal is inherently defective and readily damaged by acids, enzymes, and oral fluids. Bacteria infiltrate the resulting gaps at the composite-tooth interface and bacterial by-products demineralize the tooth and erode the adhesive. These activities lead to wider and deeper gaps that provide an ideal environment for bacteria to proliferate. This complex degradation process mediated by several biological and environmental factors damages the tooth, destroys the adhesive seal, and ultimately, leads to failure of the composite restoration. This paper describes a co-tethered dual peptide-polymer system to address composite-tooth interface vulnerability. The adhesive system incorporates an antimicrobial peptide to inhibit bacterial attack and a hydroxyapatite-binding peptide to promote remineralization of damaged tooth structure. A designer spacer sequence was incorporated into each peptide sequence to not only provide a conjugation site for methacrylate (MA) monomer but also to retain active peptide conformations and enhance the display of the peptides in the material. The resulting MA-antimicrobial peptides and MA-remineralization peptides were copolymerized into dental adhesives formulations. The results on the adhesive system composed of co-tethered peptides demonstrated both strong metabolic inhibition of *S. mutans* and localized calcium phosphate remineralization. Overall, the result offers a reconfigurable and tunable peptide-polymer hybrid system as next-generation adhesives to address composite-tooth interface vulnerability.

## 1. Introduction

Despite the extensive use of composite materials in restorative dentistry, the average clinical lifespan of these restorations is limited [1]. The high susceptibility of composite restorations to failure is a multifactorial problem involving the patient’s risk for decay, biodegradation by-products that increase the virulence of cariogenic bacteria, and gaps at the composite/tooth interface. The composite/tooth interface is initially sealed by a low-viscosity adhesive, but the fragile seal to dentin is readily damaged by acids, enzymes, and oral fluids. This damage leads to crevices that are colonized by cariogenic bacteria such as *Streptococcus mutans*. Biodegradation by-products accumulate at the dentin/adhesive (d/a) interface and increase the virulence of cariogenic bacteria, provoking a positive feedback loop that escalates the degradation.

Adhesion of the gram-positive bacterium *S. mutans*, a primary causative agent of dental caries, to the d/a interface creates an environment that promotes the attachment and growth of other bacterial species leading to the formation of a micro-ecosystem known as a biofilm [2,3,4]. In addition to causing biofilm formation, *S. mutans* produces lactic acid, which erodes the adhesive and demineralizes the adjacent tooth surface leading to permanent tissue damage [5].

Numerous bacterial-inhibition strategies including fluoride-releasing compounds [6], silver nanoparticles, zwitterionic compounds [7], and quaternary ammonium compounds (QACs) have been explored [8]. Several natural, plant-derived compounds which offer antimicrobial properties, as well as mineral deposition, have been investigated [9]. Some of these strategies have offered multi-faceted advantages such as improved adhesion, enhanced mechanical properties [10,11], and antimicrobial properties [12]. However, the vulnerability of the adhesive system remains a challenge as none of these approaches address the complex interplay of the physicochemical and mechanical factors that challenge the durability of the adhesive seal at the composite-tooth interface. Strategies to mitigate damage at these vulnerable sites must inhibit pathogenic bacterial colonization at the d/a interface, minimize bacterial drug resistance without disrupting the surrounding host-tissue viability, and promote mineralization simultaneously without provoking biocompatibility concerns. 

Antimicrobial peptides (AMPs) have attracted considerable attention due to their superior microbiocidal properties, including broad-spectrum activity and activity against biofilms, while significantly reducing the risk of bacterial drug resistance and cytotoxicity [12,13,14,15,16,17]. Moussa, et. al., has used amphipathic and antimicrobial peptides to inhibit biofilm attachment at the dentin surface [18]. A highly hydrophobic and strongly-anchored GL13K antimicrobial peptide-based coating was also shown to be a promising approach [19]. Our group has developed an antimicrobial peptide delivery system to prevent tooth decay and to reduce periodontal pathogens. We have developed antimicrobial peptides that attach to dental titanium implants to prevent infection at the implant site [20,21,22,23,24]. Similarly, our group has demonstrated the efficacy of an antimicrobial peptide, GH12, using two different approaches. In the first approach, we coupled antimicrobial peptides into the adhesive using nonbonded interactions [25]. Later, antimicrobial peptides were conjugated to the polymer network of the adhesive to enhance antimicrobial efficacy [26].

Motivated by these results, we explored a multi-factorial design to tether peptides having two discrete functions, i.e., an antimicrobial property and acting as a mineralization mediator to monomers. Copolymerization of the monomers will lead to a biohybrid polymer system that provides both antimicrobial and remineralization properties. 

Building upon the successful outcome of our previous AMP GH12 polymer conjugation, we integrated a novel antimicrobial peptide into a polymer hybrid system. We have used computationally designed and characterized antimicrobial peptide, AMP2 [27], (KWKRWWWWR) as a component of the chimeric peptide we developed to functionalize the implant surface [28]. Herein, we engineered an AMP2 derivative (AMPM7) sequence using a functional spacer for incorporation into a monomer site. This multi-factorial design also incorporated a hydroxyapatite-binding peptide (HABP) to promote remineralization of damaged mineralized tissue at the d/a interface.

In our prior work, we reported a phage display selected high-affinity HABP with the amino acid sequence of CMLPHHGAC [29]. We demonstrated that this HABP could be further engineered to design an engineered fluorescent probe to label mineralized tissues [30]. Using this HABP peptide, we developed a peptide-based approach to mediate remineralization of deficient dentin at the d/a interface [31]. The peptide-mediated approach led to calcium phosphate mineralization. 

**Figure 1:**
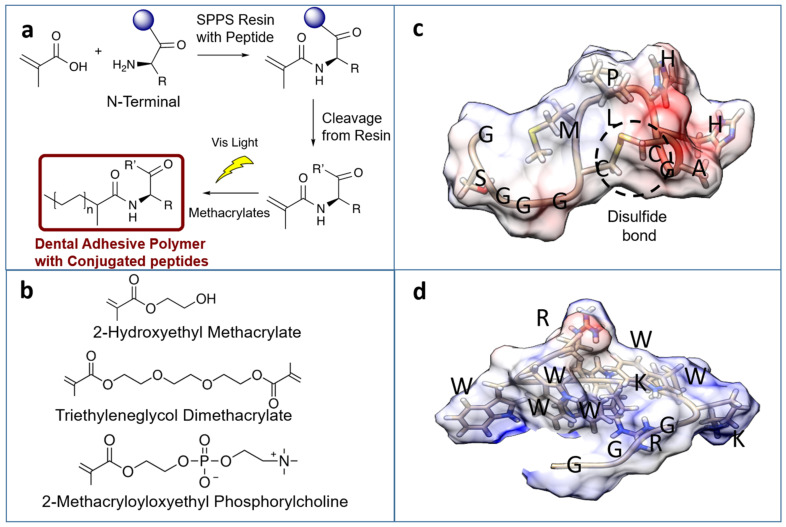
(**a**) Synthesis overview for dental adhesive polymer. (**b**) Methacrylate compounds used in dental adhesive polymer synthesis. (**c**) Bioactive peptide with HABP with spacer sequence (GSGGGCMLPHHGAC). (**d**) Bioactive peptide for AMPM7 (GSGGGKWKRWWWWR-NH2). The peptide surfaces are colored by electronegativity through the Coulombic surface tool in UCSF Chimera 1.13. Structures generated using PyRosetta folding script.

In the current study, we explore the ability to tether the mineral-binding and remineralization peptide into the polymer network to increase mineral content and nucleation at the vulnerable d/a interface. Similar to our approach for tethering AMP to polymer, we used a spacer sequence to engineer HABP derivative to conjugate it to methacrylate (MA) monomer. Both the MA-HABP and MA-AMPM7 monomers were then copolymerized into the adhesive formulation as bioactive peptide-polymer conjugates. Following polymerization, we analyzed the activity of the tunable dual peptide-polymer system containing both AMPM7 and HABP conjugates. Figure 1 provides an overview of our approach for creating this “bio-hybrid” adhesive.

Increasing the longevity of resin-based composite restorations requires an approach for transforming the fragile d/a interface into a robust, durable structure that will provide an effective barrier to decay. Our approach addresses this major need by biohybrid techniques where multifunctional bioactive molecules are combined with a state-of-the-art synthesis of novel polymers. The antimicrobial peptide and hydroxyapatite binding peptide tethered to the polymer adhesive demonstrated significant antimicrobial and mineralization activity. Our dual peptide–polymer hybrid adhesive system offers a reconfigurable approach towards overcoming challenges at the critical d/a interface.

## 2. Results and Discussion

### 2.1. Engineering Bioactive Peptides with Spacer Sequences

Bioactive peptide domains were selected to integrate specific functions into the adhesive formulations. AMPM7 was selected to provide an antimicrobial activity and HABP was selected to provide peptide-mediated remineralization as well as high mineral binding properties, as established previously within our group [21,22,24,25,26,28,29,30,31]. We included a lysine (K) at the N-terminal in each peptide to have a reactive amino group in the N(alfa) position for methacrylate (MA) monomer functionalization. In our prior studies, we have shown that incorporating a spacer domain enhanced the antimicrobial activity of a bi-functional peptide self-assembled on an implant [20,22]. Motivated by these findings, a spacer domain was incorporated between the peptide and the resin material to allow flexibility for the optimum activity. Based upon our computational analysis of the structure-function relationship for each of the peptides, the GGG and the GSGGG were used as spacer sequences for AMPM7 and HABP, respectively, in addition to K amino acid (Table S1 and Figure 1). Both AMPM7 and HABP were synthesized with the oligomeric spacers using an FMOC process. Synthesized products were verified through matrix-assisted laser-desorption ionization time-of-flight mass spectrometry (MALDI-TOF MS) (Supplementary Figures S1–S4). The calculated and observed molecular weights for the different peptides are shown in Table S1. Successful conjugation and synthesis of these peptides were confirmed by comparing the calculated and observed molecular weights. 

### 2.2. Physical Characteristics 

Polymer-only Controls: The degree of conversion of the polymer-only resin was 90.3 ± 1.5%. The water sorption profile of the polymer-only specimens reached a plateau after 24 h soaking in water at room temperature, and the final water sorption was 7 ± 1.7%. The low degree of water sorption suggests promising characteristics for the polymer-only resin. Resin formulations with a high degree of conversion and low water sorption properties help to maintain a strong bond and reduce the potential for degradation. High water sorption results in a weakening of the polymer, undermining the integrity of the adhesive bond to dentin, which subsequently allows for increased infiltration and colonization of cariogenic bacteria and a deleterious cycle of demineralization. [1,11,32].

Polymerization of Peptide-Tethered-Polymers: The polymerization kinetics was not assessed in the current study, however to date, our results [26] indicate that the curing process is not affected by the co-polymerizable peptide monomers. As an example, Young’s modulus of experimental adhesive formulations containing co-polymerizable AMP monomer was either higher or within the observed error of control adhesives [26]. The samples in our prior investigation contained the same photoinitiator system and were polymerized under conditions identical to those reported in the current study. The polymerization kinetics of peptide-tethered-polymers is part of the ongoing studies in our laboratory.

### 2.3. Effect of K-HABP on Calcium Phosphate Mineralization

**Figure 2:**
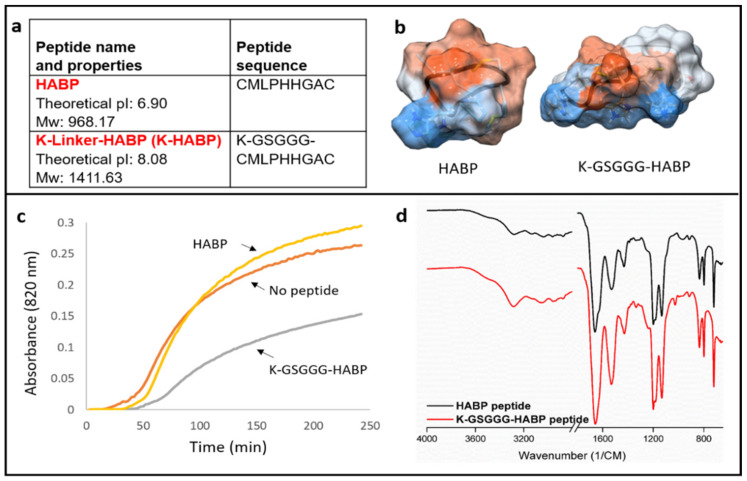
(**a**) Specifications of the peptides. (**b**) Three-dimensional molecular structure of HABP and K-GSGGG-HABP. (**c**) Optical density change during the mineralization media in the presence of HABP, K-GSGGG-HABP, and with no peptide. (**d**) The molecular structure of the peptides was confirmed using FTIR.

We performed FTIR spectroscopy on the HABP and K-GSGGG-HABP peptides and confirmed that the spectra demonstrated high similarity for both peptides (Figure 2). Both peptides contained the spectral band of 1652 cm^−1^ (Amide I) characteristic of an α-helix conformation. In addition, a spectral band of 1536 cm^−1^ was noted in the Amide II region [25,33,34,35]. The selected spacer did not cause a change in the secondary structure of the active peptide domain.

The addition of HABP increases the optical density during the mineralization reaction while the addition of K-GSGGG-HABP to the mineralization led to a decrease in optical density. The control reaction, containing no peptide, resulted in the highest rate of increase in absorbance in the remineralization solution. In the presence of 0.07 mM peptide, the increase in absorbance was reduced. A minor reduction was observed in the presence of HABP within the first 100 min (Figure 2). However, a reduction was observed in the presence of K-GSGGG-HABP at the 4 h measurement.

**Figure 3:**
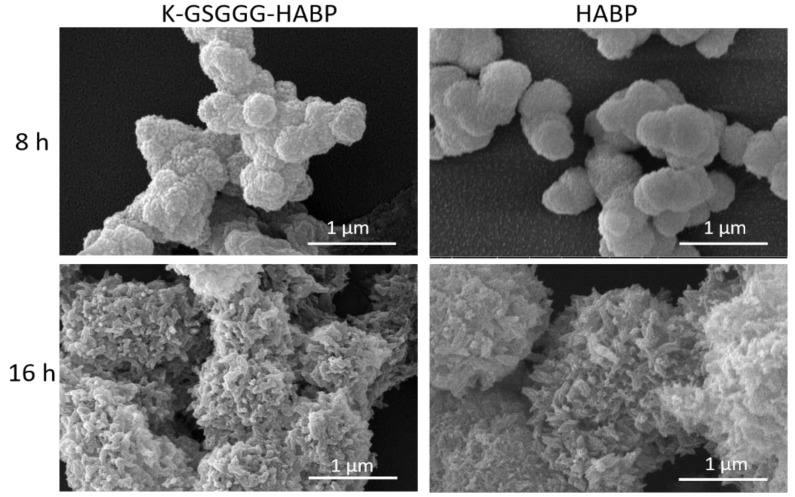
SEM images of the minerals formed in the presence of HABP and K-GSGGG-HABP.

SEM was used to compare the morphology of the particles formed in the presence of HABP and K-GSGGG-HABP peptides after 8 and 16 h incubations. The 8 and 16 h mineralization reaction samples have similar morphological characteristics (Figure 3). Spherical particles of similar shape and size were observed in both groups.

**Figure 4:**
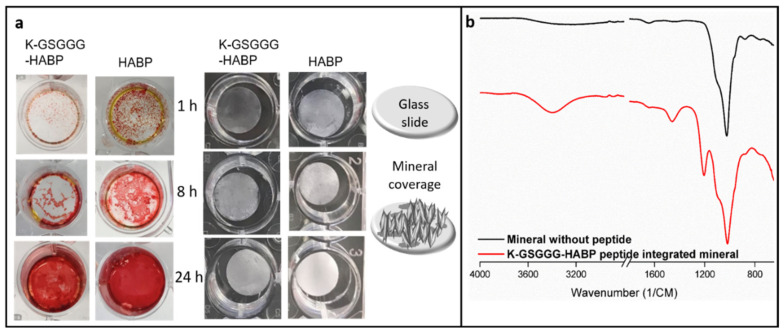
(**a**) Mineral layer obtained on the surface of the 24-well plate at different time points were stained with Alizarin red (Left), the minerals produced on the slides. The increasing mineral coverage on the glass slides over different time points (right). (**b**) FTIR results of the minerals obtained after overnight mineralization reaction with and without peptide.

In addition, following 24 h mineralization reaction, the mineral coverage on the surface became similar. Both alizarin red staining and direct mineral coverage results support this observation (Figure 4). These results indicate that K-GSGGG-HABP is a good candidate to provide the desired mineralization and adhesion to existing minerals at the d/a interface.

### 2.4. Peptide-Mediated Remineralization of the Adhesive Formulation 

There have been several attempts to increasing the remineralization of enamel caries using a variety of peptide sequences. Ding, et. al., used amelogenin-derived peptides and fluorides to remineralize artificial enamel caries [36]. In a separate study, Zheng, et. al., used a peptide with eight repetitive sequences of aspartate-serine-serine (8DSS) as a mineralization agent in a rat model [37]. A recent study incorporated a mineral-promoting peptide within a resin varnish system to enhance local remineralization on extracted primary teeth [38]. 

**Figure 5:**
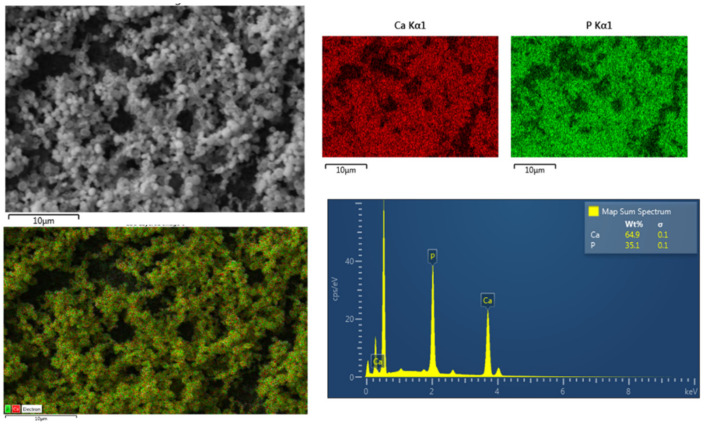
SEM image and EDX of K-GSGGG-HABP adhesive disc after mineralization.

In the current study, we used K-GSGGG-HABP peptide tethered to a dental adhesive polymer to promote peptide-mediated mineralization at the vulnerable d/a interface. The remineralization activity was assessed using an alkaline phosphatase-based mineralization assay. Following remineralization, the mineral formed on the surface of the peptide-tethered-polymer was characterized using scanning electron microscopy (SEM) with energy dispersive X-Ray spectroscopy (EDX) analysis. From the EDX analysis, the Ca/P ratio for minerals formed on the surface of the discs with tethered K-GSGGG-HABP is 1.43 (Figure 5). The Ca/P ratio can be used to identify the specific forms of calcium phosphate materials formed on the surface of the peptide-tethered polymers. The Ca/P ratios of intact enamel, dentine, and hydroxyapatite (HA) are 1.63, 1.61, and 1.67, respectively, however a drop in the local pH, commonly associated with the presence of cariogenic bacteria, can significantly impact the formed calcium phosphate phase [39,40]. In the oral cavity, calcium phosphate minerals are often derived from calcium salts binding to phosphoric acid (H_3_PO_4_), commonly referred to as calcium orthophosphates [41,42]. In general, calcium orthophosphates are highly stable and boast high solubility constants (pK_s_), critical properties associated with intact enamel, dentine, and HA [41]. In an acidic environment, the presence of different phosphate anions and available hydroxyl groups often result in CaP mineral phases with a reduced Ca/P ratio and greater water solubility, as compared to intact HA [41,42]. The mineral formed in the presence of K-GSGGG-HABP had an observed Ca/P ratio of 1.43, which falls in-between the established Ca/P ratios of 1.33 for octacalcium phosphate (OCP) and 1.50 for both tricalcium phosphates (TCP) as well as calcium-deficient hydroxyapatite (CDHA), respectively [40,42,43,44,45]. Although this Ca/P ratio is less than that of intact dentine, our tethered peptide showed the promising potential to promote intermediate phase calcium phosphate mineralization at the d/a interface.

### 2.5. Antibacterial Activity of the Polymer Discs Against S. mutans 

Minimum inhibitory concentration (MIC) was used to assess the antimicrobial activity. The lower MIC value indicates that less peptide is needed to inhibit the growth of bacteria. In our previous work, as well as several other groups, the effects of antimicrobial peptides in dental adhesive systems have been studied in detail [12,13,14,15,18,19,22,25,26,46,47,48]. In the current study, we investigated the activity of the antimicrobial peptides in the presence of the spacer sequence (KGGGKWKRWWWWR-NH2). The antimicrobial peptide without a spacer (KWKRWWWWR-NH2) had the minimum inhibitory concentration (MIC) value of 7.8 μg/mL. The spacer modification resulted in an increase in the MIC value to 15.6 μg/mL. Since this MIC value already shows a strong inhibition against *S. mutans*, this study was continued with the engineered peptide sequence design (see Supplementary Table S2 and Figure 1).

We also tested the antimicrobial activity in the presence of minerals. The mineral itself and the mineral with the K-GSGGG-HABP had no distinct antimicrobial activity with an MIC > 1000 μg/mL. Yet, when the AMPM7 is included with mineral and the mineral binding peptide, the spacer-active peptide domain showed distinct antimicrobial activity with an MIC value of 31.3 μg/mL (Figure S5). This is a significant enhancement of antimicrobial activity which was further examined using the polymer discs. 

**Figure 6:**
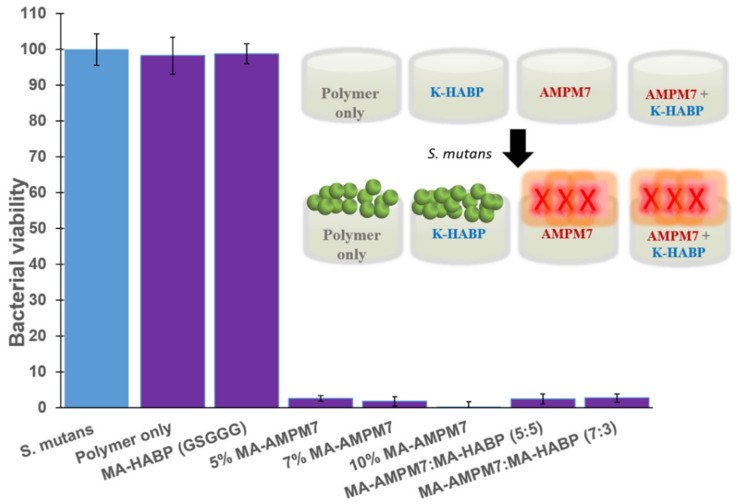
Schematic illustration and graphical representation of the viability of *S. mutans* cultures after overnight incubation with polymerized discs containing peptide samples. *S. mutans*: a positive control without a disc.

We studied the antimicrobial activity of AMP-tethered-polymer discs, HABP-tethered-polymer discs, and polymer-only controls against *S. mutans*. Neither the HABP-tethered-polymer discs nor the polymer-only controls inhibited *S. mutans* bacterial growth. In contrast, the formulations with 5 to 10 wt% AMPM7-tethered-polymer discs exhibited substantial antimicrobial activity (Figure 6). In our previous study, coupling of antimicrobial peptides in the polymer formulation without conjugation showed dependence on the presence of polar functional groups for antibacterial efficacy [25]. Here, AMPM7 has been successfully conjugated prior to polymerization and the peptide-tethered-polymer discs showed strong inhibition against *S. mutans*.

### 2.6. Dual Peptide Tethered Polymer System Offers Both Antimicrobial and Remineralization Properties 

**Figure 7:**
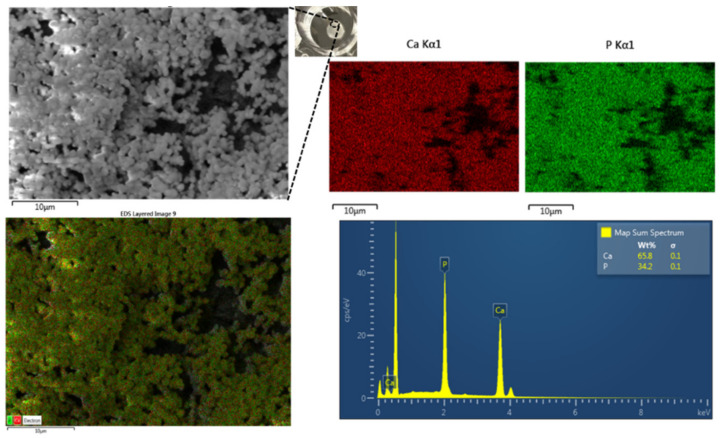
Characterization of mineralized disc surface using SEM-EDX. SEM image and EDX of K-GSGGG-HABP:AMPM7 (3:7) adhesive disc after mineralization.

We evaluated the Ca/P ratio for the mineral formed in the presence of the K-GSGGG-HABP:AMPM7-tethered-polymer discs using SEM-EDX analysis (Figure 7). When the peptides are displayed in the hybrid polymer discs with the ratio of 3:7, the Ca/P ratio for the mineral formed on the surface is 1.49, closer to the Ca/P ratio of 1.5 associated with TCP as well as that of calcium-deficient hydroxyapatite [40,41,42,45]. These results show that the mineral formed at the surface of the K-GSGGG-HABP: AMPM7 (3:7)-tethered-polymer disc has a higher Ca/P ratio (1.49) as compared to the K-GSGGG-HABP-tethered-polymer disc (Ca/P ratio 1.43). The Ca/P ratios for OCP, TCP, CDHA, and HA are 1.33, 1.5, 1.5, and 1.67, respectively [39,40,43]. Therefore, the increased Ca/P ratio observed in the dual-peptide tethered system not only offers antimicrobial properties to combat *S. mutans* but also improves the stability and decreases the solubility of the new mineral formed on the surface of the polymer. These properties allow for the peptide to remain at the interface longer and provides an opportunity to tune the interface properties. 

**Figure 8:**
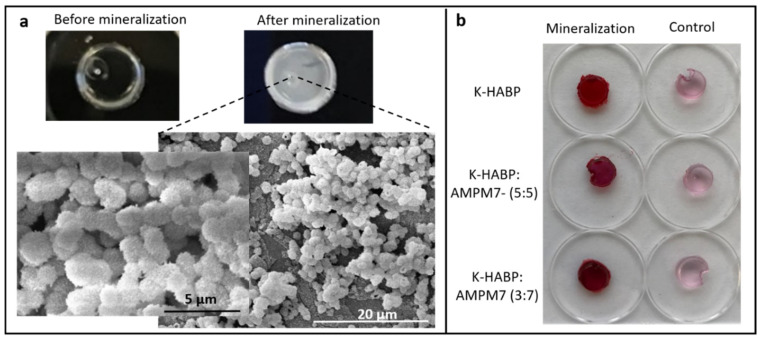
(**a**) K-GSGGG-HABP:AMPM7 integrated polymer disc samples were monitored under SEM after overnight mineralization reaction. (**b**) Peptide integrated polymer discs after mineralization were stained with Alizarin Red. The same type of discs without mineralization were used as controls.

The polymer disc samples with tethered K-GSGGG-HABP:AMPM7 peptides were next analyzed by SEM following an overnight mineralization reaction. The dual peptide tethered system showed similar mineral particle morphology as that noted with the K-GSGGG-HABP-tethered-polymer discs. The surface mineral coverage following 24h mineralization reaction was also similar (Figure 8). These results indicate that the addition of the antimicrobial peptide to form the dual-peptide tethered system did not interfere with the HABP-mediated mineral morphology. Furthermore, although the SEM images showed no morphological differences between CaP minerals formed in the presence of K-GSGGG-HABP:AMPM7 as compared to K-GSGGG-HABP, the EDX molar composition data revealed enhanced mineral stability in the dual-peptide system, supporting the synergistic effect.

## 3. Materials and Methods

### 3.1. Materials

Triethyleneglycol dimethacrylate (TEGDMA), 2-hydroxyethyl methacrylate (HEMA), 2-methacryloyloxyethyl phosphorylcholine (MPC), methacrylic acid (MA), camphoroquinone (CQ), ethyl-4-(dimethylamino) benzoate (EDMAB), diphenyliodonium hexafluorophosphate (DPIHP), dodecyltrichlorosilane, chlorhexidine digluconate (CHX), N, N-Dimethylformamide (DMF), dichloromethane (DCM) and N-methyl morpholine (NMM) were obtained from Sigma-Aldrich (St. Louis, MO, USA) and were used as received without further purification. γ‒methacryloxypropyl trimethoxysilane (MPS) was used as received from MP Biomedicals (Solon, OH, USA). Piperidine and AlamarBlue Reagent were obtained from Fisher (Thermo Fisher Scientific, Waltham, MA, USA). Rink amide resin, Fmoc-resin, Fmoc-amino acid building blocks, and 2-(1H-benzotriazole-1-yl)-1,1,3,3-tetramethyluranium hexafluorophosphate (HBTU) were purchased from AAPPTec LLC (Louisville, KY, USA). BD™ (Becton, Dickinson, and Company; Franklin Lakes, NJ, USA) Bacto™ brain heart infusion, BD BBL dehydrated brain heart infusion agar, and Corning™ (Corning, NY, USA) Clear polystyrene 96-well microplates, Corning™ 3370 were obtained from Fisher. *S. mutans* UA159 bacterial strain was from American Type Culture Collection (ATCC 700610; Manassas, VA, USA).

### 3.2. MA-Peptide and Peptide Synthesis

MA-AMPM7 and MA-HABP monomers were synthesized via an amidation reaction between the free amine group (lysine) in the N(alfa) position and the carboxylic acid group (MA). Briefly, Fmoc-resin-bound peptide with a spacer was first synthesized through Fmoc-chemistry using a solid-phase peptide synthesizer (AAPPTec Focus XC). The free amine group (lysine) in the N(alfa) position of the peptide bounding to resin was formed for next step conjugation due to the last cycle deprotection selected. Methacrylic acid, NMM, and HBTU were added to react with the Fmoc-resin-bound peptide in DMF at 23 ± 2 °C overnight under constant gentle rotation. After the conjugation reaction, the Fmoc-resin-bound product was washed sequentially with DMF, DCM, acetone, and ethanol. The crude MA-peptide monomer was then cleaved from the resins with a cocktail (81.5% TFA, 1% triisopropyl silane, 2.5% ethane dithiol, 5% thioanisole, 5% phenol, and 5% H2O) for 2-3 h at room temperature. The obtained crude MA-peptide monomer was purified on an HPLC system (Waters Corp., Milford, MA, USA) equipped with a Luna^®^ column packed with 10 µm C18 silica (250 × 4.6 mm, Phenomenex Inc., Torrance, CA, USA). The pure MA-peptide monomer was lyophilized and stored at –20 °C. Crude peptides were also cleaved from the resin in the same conditions, lyophilized, and then purified. The purified peptide fractions were combined, lyophilized, and stored at –20 °C. The molecular weight (MW) and isoelectronic point (pI) for each peptide were calculated using the ExPASy Proteomics Server.

### 3.3. Adhesive Formulations and Polymer Samples Preparation

Formulations without MA-peptide were used as the polymer-only controls. The experimental formulations contained 10 wt% MA-peptide monomers. The resin mixtures were prepared in an amber glass vial and mixed for 2 h at 23 ± 2 °C to ensure complete dissolution and promote the formation of a homogenous solution. CQ (0.5 wt%), EDMAB (0.5 wt%), and DPIHP (1.0 wt%) were used as the three-component photoinitiator (PIs) system.

### 3.4. Degree of Conversion of Polymer-Only Controls

The degree of conversion was determined using an FTIR spectrophotometer (Spectrum 400, Perkin‒Elmer, Waltham, MA, USA) in the ATR sampling model [49]. One drop (5 µL) of the polymer-only control was placed on the crystal top plate of the ATR accessory and covered with a mylar film. After 30 spectra were collected, the resin was light irradiated for 40 s using a commercial light source (Spectrum^®^ 800) with an intensity of 550 mW/cm^2^ [10]. The double bond conversion was monitored by the band ratio profile-1637cm^−1^ (C=C)/1710 cm^−1^ (C=O). The average of the last 30 values of the time-based spectra was reported as the degree of the conversion value. Three measurements were recorded for each control.

### 3.5. Water Sorption of Polymer-Only Controls

The experimental protocol for the water sorption analyses has been reported [50]. Five disc-shaped samples (Tzero^®^ Hermetic lid; TA Instruments, New Castle, DE, USA) were prepared and post-cured at room temperature for 48 h. The samples were prewashed in distilled water for five days to remove the unreacted monomer. Next, the specimens were dried in a convection oven at 37 °C for two days and then stored in a vacuum oven in the presence of freshly dried silica gel. The samples were removed every 24 h and weighed using a calibrated electronic balance (resolution of 0.01 mg, Mettler Toledo, XS205 Dual range, Columbus, OH, USA). This process was continued until a constant mass (m_1_) was recorded for each specimen. The dried specimens were immersed in distilled water and they were removed at fixed time intervals (3, 6, 24, and 48 h), blotted to remove excess water, weighed (m_2_), and returned to the water until a constant weight was obtained. The value (%) for water sorption (W_sp_) was calculated by the following equation:

(1) $$W_{sp}=\frac{m_{2}-m_{1}}{m_{2}}\times 100\%,$$

### 3.6. Specimen Preparation for Antimicrobial and Mineralization Tests

Round discs (4 mm diameter and 1.2 mm thick) were used to perform the antimicrobial activity and mineralization tests. The prepared resins were injected into a Tzero^®^ Hermetic Lid (P/N: 900797.901) or Tzero^®^ low mass pan (P/N: 901683.901), covered with a dodecyltrichlorosilane-modified glass cover (22 mm × 30 mm, Fisherfinest^®^), then light-cured for 40 s at 23 ± 2 °C using a commercial visible light lamp (Spectrum^®^ 800, Dentsply, Milford, DE, USA. Intensity is 550 mW/cm^2^) [10]. The polymerized samples were stored in the dark at 23 ± 2 °C for at least 48 h before testing.

### 3.7. Mineralization

A mineralization solution containing 24 mM Ca^2+^ (CaCl_2_) and 14.4 mM β -Glycerophosphate in 25 mM Tris-HCl buffer (pH 7.4) was used for mineralization studies. To compare the effect of HABP and K-HABP on calcium phosphate nucleation, an alkaline phosphatase (AP) based mineralization system was used. AP (FastAP, Fermentas; Waltham, MA, USA) was used to start the mineralization reaction. The reactions were carried out at 37 °C. Mineralization solutions containing 0.07 mM HABP and K-HABP peptides were prepared in a 96-well plate. The mineralization solution without peptide was used as the control sample. The mineral formation was monitored by measuring absorbance at 820 nm wavelength using a microplate reader (Cytation3, Biotek; Winooski, VT, USA). 

Additionally, mineralization reaction has also been run in a 24-well plate. Following the mineralization reactions in a 24-well plate, calcium phosphate layers were formed on glass cover slides and samples were washed twice with deionized water. Upon completion of each time interval, the glass slides were removed and the areas covered by minerals were analyzed.

### 3.8. Fourier-Transform Infrared Spectroscopy (FTIR)

The molecular structures of the synthesized peptides and minerals were confirmed using FTIR (Spectrum 400, Perkin-Elmer, Waltham, MA, USA), equipped with an attenuated total reflectance (ATR) accessory (PIKE Technologies Gladi-ATR, Madison, WI, USA) at a spectral resolution of 4 cm^−1^.

### 3.9. Alizarin Red Staining

The formation of calcium phosphate layers on the surface of the 24-well plate were determined by alizarin red staining. Mineralization solutions containing 0.07 mM HABP and K-HABP peptides were prepared in a 24-well plate. The mineralization solution without peptide was prepared as the control sample. The mineralization solution was removed at designated time points and the minerals on the surface were washed twice with deionized water. Thereafter, the water was removed and 1 mL of alizarin red solution was added to each well for 15 min. The wells were then washed with water to remove excess dye. The stained calcium phosphate layers were imaged. Mineralized polymer discs were also stained with alizarin red.

### 3.10. Scanning Electron Microscopy (SEM)

20 μL of the mineralization reaction solution was placed onto the glass slide. Following 5 min of incubation, the remaining liquid was carefully removed using a fiber optic cleaning wipe. After drying, samples were stored in a desiccated container. SEM imaging was performed at 10 kV using FEI Versa 3-D SEM.

### 3.11. Antimicrobial Activity Assays

Inhibitory concentration assays were conducted using a standard broth microdilution method, according to Clinical and Laboratory Standards Institute (CLSI) protocol [47,51]. Briefly, two-fold serial dilutions were prepared with sterile deionized water to achieve concentrations ranging from 5 to 1250 μg/mL in a volume of 20 μL, after which 80 μL of BHI broth and 100 μL of bacterial culture containing 2 × 10^4^ CFU/mL were added. Thus, each well-contained peptide or MA-peptide monomer concentrations ranging from 0.5 to 125 μg/mL and a final bacterial concentration of 1 × 10^4^ CFU/mL. The well that exclusively contained *S. mutans* served as the negative control for bacterial growth/negative control for antibacterial activity. The positive control for bacterial growth/positive control for antibacterial activity was identical to the negative control, except that the 20 μL H_2_O was replaced with 20 μL of 63 μg/mL CHX solution. The blank well contained 20 μL H_2_O and 180 μL medium without cells. The 96-well plate was incubated in the presence of 5% CO_2_ at 37 °C overnight. The solution of each well was transferred to a new plate. The bacterial cell viability was assessed by fluorescence intensity using an established AlamarBlue assay [52,53]. The minimum inhibitory concentration (MIC) value was defined as the lowest peptide or MA-peptide monomer concentration corresponding to the zero-value measured via AlamarBlue assay. Experiments were repeated three times per specimen treatment and concentration.

In-solution antimicrobial activity of peptide conjugated resin discs was assessed. The round, disc polymer specimens were soaked in water for five days to remove unreacted components [54]. The polymer specimens were subsequently assessed for in-solution antimicrobial activity. Briefly, the soaked disc samples were blotted to remove excess water and one disc was transferred to the microwell in a 96-well plate. Following, 20 μL H_2_O, 80 μL of BHI broth, and 100 μL of BHI broth containing 2 × 10^4^ CFU/mL *S. mutans* cells were added to each well to a final bacterial concentration of 1 × 10^4^ CFU/mL. After overnight incubation in the presence of 5% CO_2_ at 37 °C, the solution of each well was transferred to a new plate, and AlamarBlue dye was added, following the manufacturer‘s protocol. Samples were incubated for 2 h before fluorescence intensity was measured at Ex565/Em595. The well that contained *S. mutans* served as the negative control. Polymer-only demonstrates the antibacterial activity of the system without MA-peptide monomer in the formulation. The blank well contained 20 μL H_2_O and 180 μL medium without cells. Experiments were repeated a minimum of three times per specimen treatment.

## 4. Conclusions

The average clinical lifespan of the composite dental restorations is limited and recurrent decay at the composite/tooth margin where the adhesive is applied is the primary reason for the short clinical lifespan. In this paper, we address this composite/tooth-interface vulnerability through multi-faceted approaches that remineralize damaged dentin and inhibit bacterial attack. Our design incorporates dual peptide systems into our polymer―an antimicrobial peptide to specifically inhibit *S. mutans* bacterial attack and a hydroxyapatite-binding peptide to promote remineralization of damaged tooth structure. Our novel engineered-peptide design strategy includes a spacer that provides reactive groups to enable simultaneous tethering of both functionally distinct peptides to the monomer as well as providing the length and flexibility required to maintain the peptide’s original bioactivities when applied as a “bio-hybrid” adhesive at the tooth surface. Building upon the promising dual peptide-tethered-polymer system, future studies must address aspects such as the retention of the antimicrobial activity over a longer time period and under relevant in vivo conditions. The delivery of bioactive peptides tethered to polymers provides the opportunity to develop reconfigurable peptide-polymer hybrid dental adhesive systems. Such a system can be tunable depending on the clinical need. The results offer a reconfigurable, co-tethered dual peptide-polymer system as next-generation adhesives to address composite-tooth interface vulnerability.

## Data Availability

Data is contained within the article or Supplementary Materials.

## Supplementary Materials

The following are available online at https://www.mdpi.com/article/10.3390/ijms22126552/s1.
