# Supplementary material for: Reconfigurable Dual Peptide Tethered Polymer System Offers a Synergistic Solution for Next Generation Dental Adhesives
Source: Int J Mol Sci. 2021 Jun 18;22(12):6552. doi: 10.3390/ijms22126552 (PMC8235192; doi:10.3390/ijms22126552)
Supplement: Supplementary file 1 [file ijms-22-06552-s001.zip › ijms-1251912-supplementary.pdf]

Reconfigurable Dual Peptide Tethered Polymer System Offers a Synergistic Solution for Next Generation Dental Adhesives

Esra Yuca[1], *Sheng-Xue Xie*[2], *Linyong Song*[3], *Kyle Boone*[4], *Sarah K. Woolfolk*[5], *Nilan J.B. Kamathewatta*[6], *Philip Elrod*[7], *Paulette Spencer*[8], *and Candan Tamerler*[9, *]

[1] IBER Institute for Bioengineering Research, University of Kansas (KU), 1530 W. 15th St, Lawrence, KS 66045, USA; Department of Molecular Biology and Genetics, Yildiz Technical University, Istanbul, 34210, eyuca@yildiz.edu.tr
[2] IBER Institute for Bioengineering Research, University of Kansas (KU), 1530 W. 15th St, Lawrence, KS 66045, USA; sxie@ku.edu
[3] IBER Institute for Bioengineering Research, University of Kansas (KU), 1530 W. 15th St, Lawrence, KS 66045, USA; leonsong@ku.edu
[4] IBER Institute for Bioengineering Research, University of Kansas (KU), 1530 W. 15th St, Lawrence, KS 66045, USA; k097b443@ku.edu
[5] IBER Institute for Bioengineering Research, Bioengineering Program, University of Kansas (KU), 1530 W. 15th St, Lawrence, KS 66045, USA; sarah.vanoosten@ku.edu
[6] IBER Institute for Bioengineering Research, University of Kansas (KU), 1530 W. 15th St, Lawrence, KS 66045, USA; nilan_jayabahu@ku.edu
[7] Bioengineering Program, University of Kansas (KU), 1530 W. 15th St, Lawrence, KS 66045; philip.m.elrod@ku.edu
[8] IBER Institute for Bioengineering Research, Department of Mechanical Engineering, Bioengineering Program, University of Kansas (KU), 1530 W. 15th St, Lawrence, KS 66045, USA; pspencer@ku.edu
[9] IBER Institute for Bioengineering Research, Department of Mechanical Engineering, Bioengineering Program, University of Kansas (KU), 1530 W. 15th St, Lawrence, KS 66045, USA; ctamerler@ku.edu

* Correspondence: Candan Tamerler; ctamerler@ku.edu

Supplemental Table S1. Molecular weights of peptides synthesized and peptide-monomer conjugates with sequences.

Peptide Conjugate	Sequence	Calculated MW (Da)	Observed MW (Da)
K-GSGGGK-HABP	K-GSGGG-CMLPHHGAC	1411.6	1411.1
MA-K-GSGGG-HABP	MA-K-GSGGG-CMLPHHGAC	1479.7	1479.5
AMPM7	K-GGG-KWKRWWWWR-NH2	1816.1	1816.0
MA-AMPM7	MA-K-GGG-KWKRWWWWR-NH2	1884.2	1884.1

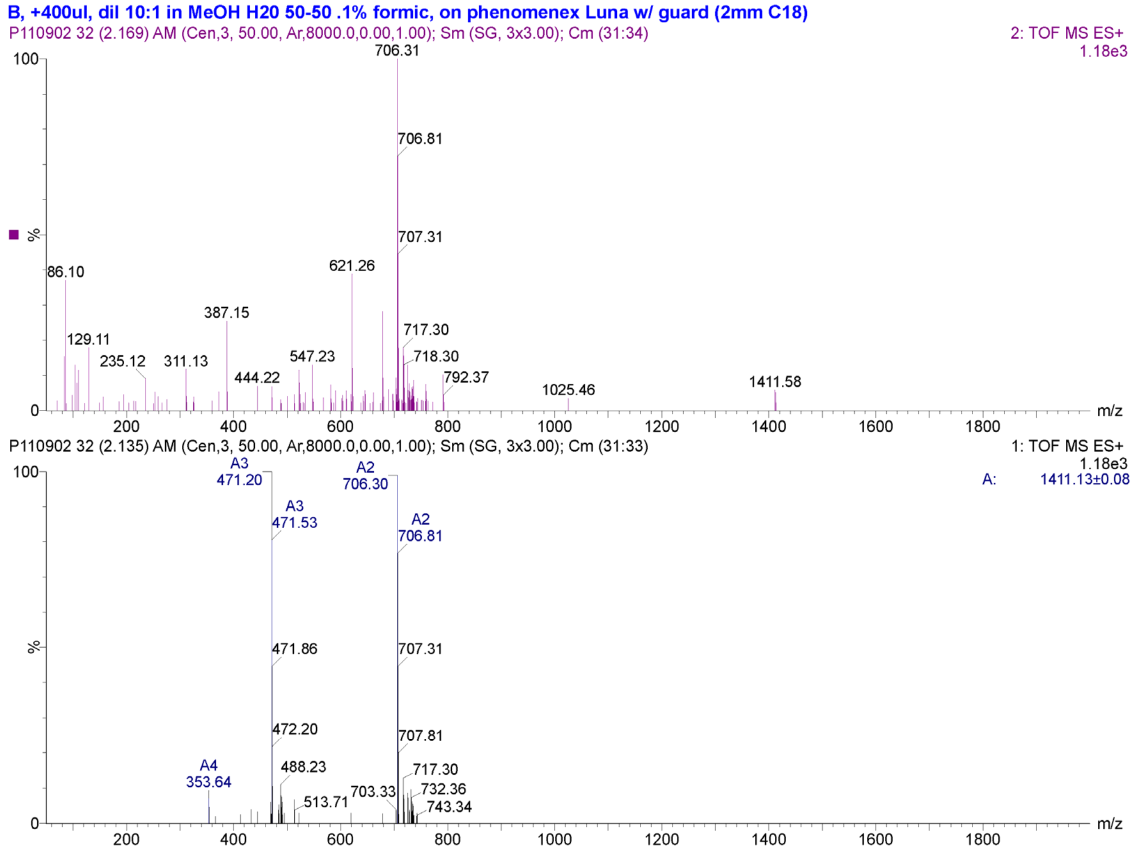

Supplemental Figure S1. MALDI-TOF MS results for K-GSGGGK-HABP

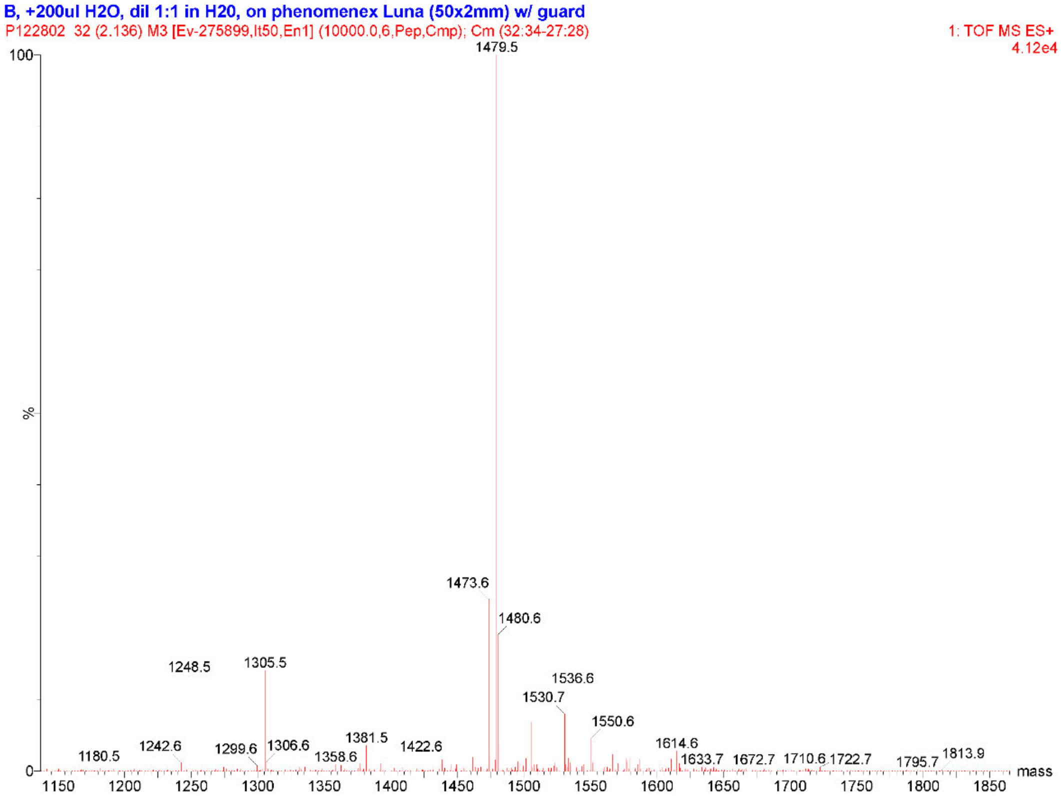

Supplemental Figure S2. MALDI-TOF MS results for MA-K-GSGGGK-HABP

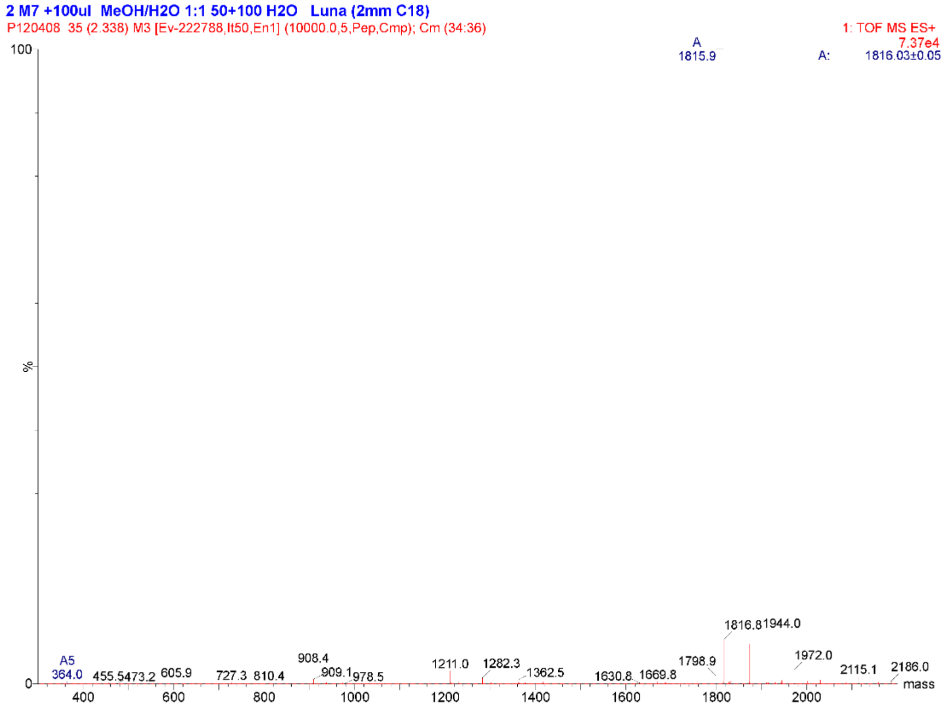

Supplemental Figure S3. MALDI-TOF MS results for AMPM7

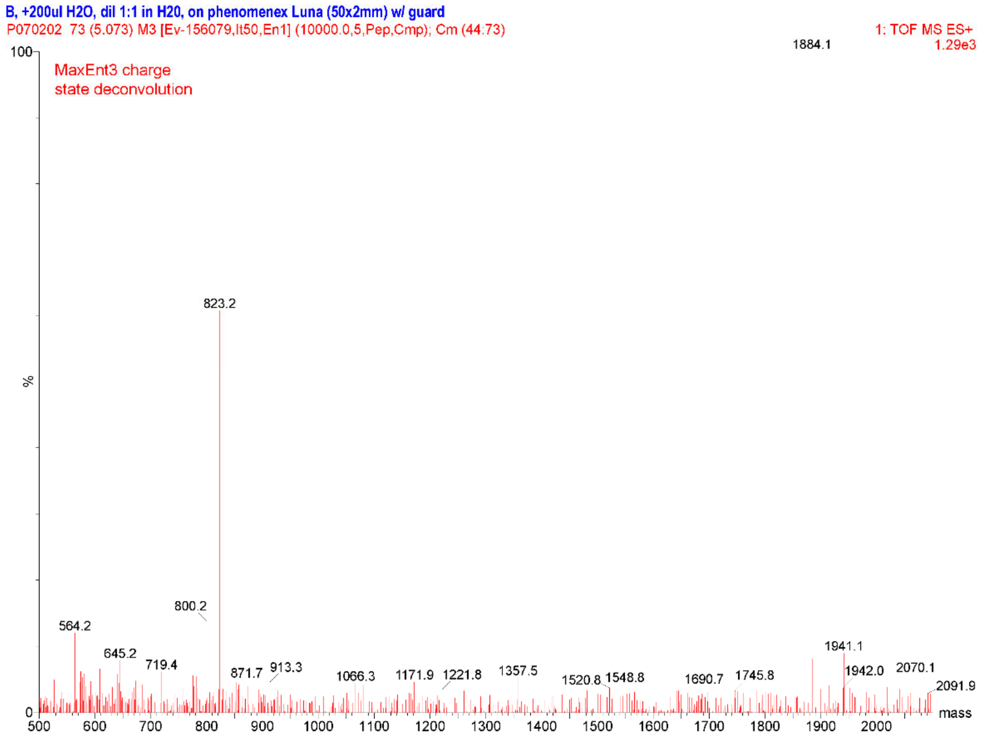

Supplemental Figure S4. MALDI-TOF MS results for MA-AMPM7

Supplemental Table S2. Minimum inhibitory concentration (MIC) of peptides synthesized and peptide-monomer conjugates against 10^4 CFU *S. mutans*.

Peptide	Sequence	MIC (µg/mL)
AMPM7	KGGGKWKRWWWWR-NH2	7.8
MA-AMPM7	MA-KGGGKWKRWWWWR-NH2	15.6
MA-K-GSGGG-HABP	MA-KGSGGGCMLPHHGAC	>250
MA (monomer)	C4H6O2	>1250

Sample	MIC (μg/mL)
Mineral Control	>1000
K-GSGGG-HABP integrated mineral	>1000
AMPM7+ K-GSGGG-HABP integrated mineral	31.3

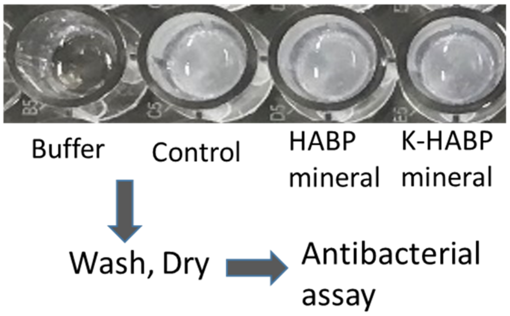

Supplemental Figure S5: Activity of dental adhesive components and mineral against *S. mutans* at 10^4 CFU/mL
